# Animals as Sentinels of Bioterrorism Agents

**DOI:** 10.3201/eid1204.051120

**Published:** 2006-04

**Authors:** Peter Rabinowitz, Zimra Gordon, Daniel Chudnov, Matthew Wilcox, Lynda Odofin, Ann Liu, Joshua Dein

**Affiliations:** * Yale University School of Medicine, New Haven, Connecticut, USA;; † Rippowam Animal Hospital, Stamford, Connecticut, USA;; ‡ US Geological Survey National Wildlife Health Center, Madison, Wisconsin, USA

**Keywords:** bioterrorism, animals, sentinel surveillance, evidence based medicine, Zoonoses, biological warfare

## Abstract

We conducted a systematic review of the scientific literature from 1966 to 2005 to determine whether animals could provide early warning of a bioterrorism attack, serve as markers for ongoing exposure risk, and amplify or propagate a bioterrorism outbreak. We found evidence that, for certain bioterrorism agents, pets, wildlife, or livestock could provide early warning and that for other agents, humans would likely manifest symptoms before illness could be detected in animals. After an acute attack, active surveillance of wild or domestic animal populations could help identify many ongoing exposure risks. If certain bioterrorism agents found their way into animal populations, they could spread widely through animal-to-animal transmission and prove difficult to control. The public health infrastructure must look beyond passive surveillance of acute animal disease events to build capacity for active surveillance and intervention efforts to detect and control ongoing outbreaks of disease in domestic and wild animal populations.

## Abstract 2

Pets, wildlife, or livestock could provide early warning.

Most priority bioterrorism agents are zoonotic in origin. As a result, an attack on human populations with a bioterrorism agent would likely pose a health risk to animal populations in the target area; therefore, integrating veterinary and human public health surveillance efforts is essential. The Centers for Disease Control and Prevention (CDC), in planning for the early detection and management of a biological terrorism attack, has recommended the "prompt diagnosis of unusual or suspicious health problems in animals," as well as establishing "criteria for investigating and evaluating suspicious clusters of human and animal disease or injury and triggers for notifying law enforcement of suspected acts of biological or chemical terrorism" (*1*). Similarly, an indicator of a biological terrorism attack would be "increased numbers of sick or dead animals, often of different species. Some BW (biological warfare) agents are capable of infecting/intoxicating a wide range of hosts" (*2*). In part because of such recommendations, calls have been made for enhanced veterinary surveillance for outbreaks of animal disease caused by bioterrorism agents and better communication between animal health and human health professionals. For such efforts to succeed, the relevance to human health of disease events in animals must be established. The potential use of animals as "sentinels" of a human bioterrorism attack can be differentiated from the possibility of a direct attack on animals of agricultural importance (agroterrorism) and is the subject of this review.

First, animals could provide an early warning to humans if clinical signs could be detected before human illness emerged or soon enough to allow preventive measures to be initiated. This early detection could occur because an animal species had increased susceptibility to a particular agent, because the disease caused by the agent had a shorter incubation period, or because animals were exposed sooner (or at more intense and continuous levels) than the human population (*2*). The simultaneous appearance of disease signs and symptoms in animals may contribute to the more rapid identification of a biological warfare agent that was producing nonspecific effects in nearby persons.

Second, if a released biological agent persists in the environment (such as soil, water, or air), active surveillance for sporadic illness in animals could help detect ongoing exposure risks. Additionally, the geographic pattern of sick or dead animals could indicate the persistence of a biological threat (*2*).

Finally, animal populations such as wild birds, commercially shipped livestock, and animals involved in the local or international pet trade, could play a role in the maintenance and spread of an epidemic attributable to an intentional release of a biological agent. Detecting the agent in such mobile populations could therefore signal the ongoing spread of the agent and provide an opportunity for interventions to prevent further spread.

Previous reviews have discussed the implications of bioterrorism attacks on human and animal health (*3*). Yet these reviews did not examine the strength of evidence or attempt to determine whether animals could be effective sentinels for particular agents.

We therefore reviewed the biomedical literature for evidence that animals could fulfill the above criteria for sentinel potential. We also hypothesized that large gaps in knowledge exist in this area, including different levels of evidence regarding specific agents and types of animals.

## Methods

We systematically searched the biomedical literature from 1966 to 2005 for reports of adverse health events in animals that were attributed to potential bioterrorism agents. The CDC publication Biological and Chemical Terrorism: Strategic Plan for Preparedness and Response (*1*) contains a list of biological and chemical agents that could be used in a terrorist attack. Infectious agents are categorized as A, B, or C, depending on their risk to public health. We searched the Medline database for reports of animal exposure to these biological agents. As a further check, we performed focused searches for individual agents in the CAB Abstracts and Agricultural Online Access (AGRICOLA) databases and also reviewed the bibliographies of recent bioterrorism reviews to locate additional sources.

Our search method used both the name of the agent and the terms "animals, wild" "animals, domestic" and "animals, zoo." For each agent, we searched for peer-reviewed studies of infection in animal populations caused by a specific agent, as well as authoritative subject reviews. The episodes of infection included both actual bioterrorism events as well as naturally occurring epizootics. This search process identified ≈6,000 potential citations, including original journal articles, textbook chapters, and reviews, which were manually culled for relevance to animal sentinel issues; this process resulted in ≈200 citations available for final analysis.

Studies that included data about relative incubation periods and susceptibilities in animals were compared to human data to determine whether evidence supported the use of animals as early warning of bioterrorism agents. We also included in this category reports of animals displaying evidence of infection before nearby human populations did. Data on human incubation periods and infective doses for individual agents were obtained from standard references on biological warfare and terrorism (*4*). Studies that detected symptomatic infection or biomarkers of infection for agents that persist in the environment were reviewed to determine whether they supported the utility of animals for ongoing exposure monitoring. Studies that demonstrated a substantial degree of animal-to-animal or animal-to-human transmission (with or without a vector) were considered to provide evidence that animals could propagate an outbreak of infectious disease caused by a deliberately released pathogen.

### Analysis of Evidence

Studies located in the above search were classified according to agent, disease, species, and study method, and these data were then entered into an online database of animals as sentinels of human environmental health hazards (available from http://canarydatabase.org/) for further analysis. For the purposes of this review, we created a taxonomy for evidence regarding animals of sentinels based on existing evidence-based medicine taxonomies that provide guidelines for assigning levels of evidence based on quality and consistency of scientific studies (*5*). We considered level 1 evidence studies to include experimental studies, cohort studies, and systematic reviews of high-quality studies with consistent findings. We classified case-control studies and cross-sectional surveys of animals as level 2 evidence. Evidence from professional consensus statements, textbooks, and descriptive case reports was classified as level 3 evidence. To arrive at an overall strength of recommendation based on a body of evidence, we used these levels of evidence to determine the overall strength of the recommendation that animals could serve in the 3 sentinel capacities of early warning signal, ongoing exposure indicator, and potential propagator and spreader of a bioterrorism agent.

## Results

The Table displays the evidence found for animals serving in a sentinel capacity for specific agents or classes of agents.

**Table:** Evidence for animals as sentinels of bioterrorism agents*

Agent/disease	Animals provide early warning of acute bioterrorism attack	Animals could be markers for ongoing exposure risk	Animals can propagate/maintain epidemic
Category A			
	Anthrax	Yes: sheep, cattle (level 3 evidence [6]) No: dogs and pigs (level 1 evidence [7])	Yes: sheep, cattle (level 3 evidence [6,8])	–
	Plague	Yes: cats (level 1 evidence [9])	Yes: dogs, cats (level 1 evidence [9]), multiple species (level 2 evidence [10])	Yes: cats, camels, goats (level 3 evidence [11,12])
	Tularemia	No (level 3 evidence [13])	Yes: rodents (level 2 evidence [14]) No: horses, cows (level 2 evidence [13])	Yes: ticks, rodents, prairie dogs (level 2 evidence [15])
	Botulism	No (level 3 evidence [16])	No (level 3 evidence [16])	No (level 3 evidence [16)
	Filovirus infection	–	–	Yes: wildlife (level 3 evidence [17])
Category B			
	Q fever	No: sheep (level 1 evidence [18])	Yes: wild hogs, goats (level 2 evidence [19,20])	Yes: cats, sheep, goat, cattle (level 3 evidence [21])
	Brucellosis	No (level 3 evidence [3])	Yes: cattle (level 2 [22])	Yes: wildlife, cattle, dogs (level 3 evidence [23])
	Foodborne illness: *Salmonella* spp; *Shigella* spp.; *Cryptosporidium* spp, etc.	Yes: cattle (level 3 evidence [24])	–	–
	Glanders	–	Yes: horses (level 2 evidence [25])	Yes: horses (level 3 evidence [26])
	Alphaviruses (VEE/EEE)	Yes: horses (level 3 evidence [26]))	Yes: birds (level 1 evidence [27])	Yes: wild birds (level 2 evidence [27])
	Rift valley fever	Yes: cattle, sheep (level 3 evidence [23])	Yes: sheep (level 1 evidence [28])	Yes: mosquitoes, rodents (level 1 evidence [29])
	Ricin toxin	–	–	–
	Epsilon toxin	–	–	–
Category C (emerging diseases)			
	Nipah virus	–	Yes: multiple species (level 3 evidence [30])	Yes: pigs (level 1 evidence [31])
	Hantavirus	No (level 2 evidence [32])	Yes: multiple species (level 2 evidence [32])	Yes: rodents (level 2 evidence [33])
	Flavivirus (WN, JE)	Yes: wild birds (level 3 evidence [34])	Yes: mosquitoes, birds (level 2 evidence [35])	Yes: birds (level 1 evidence [36])

*Level 1 evidence, experimental or cohort study or randomized clinical trial; level 2 evidence, case-control or cross-sectional study; level 3 evidence, case reports or case series, expert opinion; .–, insufficient evidence found; VEE/EEE, Venezuelan equine encephalitis /eastern equine encephalitis; WN, West Nile; JE, Japanese encephalitis.

### Evidence That Animals Provide Early Warning of an Acute Bioterrorism Attack

For a number of agents, this review found evidence that animals might be affected before human populations. For *Bacillus anthracis*, whether animals would have a shorter incubation period in the event of an aerosol release was not clear, since the incubation period in the 2001 mail attacks was <4 days, while during the 1979 release of *B*. *anthracis* from a Soviet military laboratory, human symptoms began in 2 days, with death in as few as 6 days (*6*). At the same time, while human cases in Sverdlovsk were concentrated along the path of the prevailing wind <4 km from the laboratory, livestock, including sheep and cows, began dying 3 days after the release in 6 villages located along the path of the aerosol at a distance <50 km downwind from the facility. No human cases were reported in these villages. Calculations of the airborne *B*. *anthracis* dosage at a town where several sheep and a cow died indicate that the animals were exposed to a dose more than an order of magnitude lower than humans received near the weapons facility. This finding suggests that sheep and cows are more susceptible than humans, although they could have also remained outside in the path of the aerosol for a longer period, which led to greater exposure (*6*).

For *Yersinia pestis*, evidence from experimental inhalation studies in cats indicates that the usual incubation period for symptoms of plague to develop after an inhalation exposure may be shorter (1–2 days) than the presumed incubation time for humans (1–6 days), which provides evidence that symptoms develop in cats at the same time as in humans, and thus may have some sentinel value. In contrast to the findings for anthrax and plague, however, we were unable to find evidence that animals could provide early warning of infection with airborne *Francisella tularensis*. During a prolonged outbreak of pneumonic tularemia in Scandinavia, for example, febrile illness developed in a number of horses, a cow, and a pig, but apparently not before the onset of disease in humans living nearby (*13*).

For foodborne illnesses, including botulism, animals would likely not manifest illness before humans if an attack were directed at humans, since in a typical attack scenario, food would be infected during the distribution pathway before consumption by humans and not necessarily allow for animal consumption before this. We did not locate reports of animals becoming symptomatic with foodborne illness before the onset of human cases. However, if the attack on the food supply were directed at the animals themselves, they could potentially manifest symptoms before humans who would consume the meat, eggs, or dairy products (*24*). Attacks on water supplies with agents such as *Clostridium botulinum* could put humans at risk as well, although dilution and water treatment would reduce the risk (*37*). An exception may be *Cryptosporidium* spp., which have caused widespread outbreaks through the water supply. Waterfowl die-offs from type C and type E botulism have been well documented, although these types are not well recognized as causes of clinical *C*. *botulinum* poisoning in humans, but the fact that primates are susceptible to type C makes *C*. *botulinum* poisoning a possibility. On the whole, however, an attack on human populations with *C*. *botulinum* would probably not be first detected in animals; the illness would have such a short incubation period in humans that they would become symptomatic at the same time as or before the animals.

For alphaviruses, natural outbreaks have often appeared in animal populations before they affected humans, for example, eastern equine encephalitis virus often appears in equines 2 weeks before humans become symptomatic (*28*). Whether the same pattern would hold true during an attack with an aerosol is not clear. For certain newer agents, such as filoviruses and Nipah virus, current evidence is insufficient to state whether after a generalized release of an aerosolized agent, animal infection would precede that in humans. Studies in Africa have demonstrated that Ebola virus outbreaks can be preceded by deaths in primates as well as in other animal species such as duikers (type of antelope) (*17*), but whether a generalized attack that used Ebola virus in the United States would affect certain animal species first is unknown.

For a number of agents, including *Brucella* spp., *Coxiella burnetii*, and hantavirus, infection in animals is either asymptomatic or develops so slowly that recognizable human cases seem certain to precede animal cases if the agents are released as an aerosol. Finally, the illnesses caused by some agents appear to have shorter incubation times in animals, for example, the 12-hour incubation period for Rift Valley fever in calves and lambs (*23*) compared to the incubation period of several days in humans.

### Evidence That Animals Could Be Markers for Ongoing Exposure Risk

After the acute release of a bioterrorist agent, public health officials could be faced with the possibility of an agent persisting in the environment. Anthrax spores can survive for years in soil. Therefore, monitoring for sporadic cases in animal populations such as livestock could indicate so-called exposure hot spots. Although dogs and cats are less susceptible to *B*. *anthracis* than ruminants, their proximity to humans and their contact with soil could make them sentinels; for example, anthrax developed in a Labrador retriever after the dog hunted in a freshly plowed field (*8*). In the case of ongoing exposures to an agent that has become established in an animal population, case detection could be useful; this situation has been seen for plague in cats (*9*). Similarly, agents like *Brucella* spp. and *C*. *burnetii*, although they do not cause severe acute illness in animals, could be detected by recognizing increased rates of abortion among a variety of species.

Aside from case detection, active surveillance with surveys of animals may be useful; this surveillance may require testing wildlife as well. Such testing could involve antibody seroprevalence or use of polymerase chain reaction techniques to detect antigen. Evidence about the usefulness of such an approach was inconsistent. During an epidemic of pneumonic tularemia, attributable to contaminated hay, the etiologic agent, *F*. *tularensis*, can persist in the environment; a serosurvey of asymptomatic livestock (horses and cows) did not show evidence of exposure (*13*). By contrast, in a more recent tularemia outbreak, serosurveys in the wildlife population did show antibodies in a skunk and a rat that lived near persons who had become infected after mowing fields (*14*).

### Evidence That Animals Could Propagate an Epidemic Caused by a Bioterrorism Agent

A number of biological terrorism agents have little potential for secondary spread in either animal or human populations, including *B*. *anthracis* and *C*. *botulinum*. For other agents, however, we found evidence that their introduction into an animal population could cause an epizootic that would then place additional human populations at risk. For example, studies of mosquitoes native to the United States have demonstrated their potential to spread a disease such as Rift Valley fever through livestock and other animal populations (*29*), even though person-to-person transmission does not occur. The results of animal surveillance for Ebola virus in Africa found that ongoing outbreaks in both primates and duikers suggest that the virus may be able to propagate in a wildlife population (*17*), however, this characteristic has not been demonstrated in US wildlife species.

Agents such as *C*. *burnetii* and *Brucella* spp. spread easily in animal populations through direct contact and can then pose a wider risk to humans, even though human-to-human transmission does not occur. Agents such as alphaviruses that are prevalent in wild bird populations can spread over a wide area in a short time (*27*). Experimental studies have documented that viruses such as West Nile virus can easily spread from animal to animal in bird populations (*36*).

## Discussion

For a number of biological terrorism agents, we found evidence that animals could provide early warning of an acute attack. For the agents for which we found evidence of sentinel potential, a key factor was the relative exposure risk of an animal compared to that of a nearby human population. However, in an actual event involving both humans and animals, the fact that disease was detected sooner in animals could be due to an interplay of a number of factors, including local infrastructure of animal and human health services, public awareness, and laboratory capacity. For other agents, however, humans would demonstrate symptoms at the same time as nearby animals or before. Therefore, the strength of evidence regarding animals serving as early indicators of an attack depends strongly on the agent and species involved. For some agents for which animals would not provide early warning, however, animals could help detect pockets of ongoing exposure risk. For the remainder of agents, evidence regarding the value of animals as sentinels is insufficient at this time.

Overall, according to our classification taxonomy, the strength of the recommendation that animals could provide early warning of an acute bioterrorism attack seems to be, at best, "fair" because of the inconsistency of the evidence. A somewhat more consistent level of evidence appeared to support the recommendation that animals could be markers for ongoing exposure risk and also that animals could play a strong role in propagating outbreaks caused by particular agents. At the same time, our ability to assess the overall strength of evidence for such recommendations was hampered by large gaps in current knowledge.

These findings suggest the need for certain steps related to preparedness for biological agent attacks. First, improved communication is needed between animal health and human health professionals, so that sentinel events could be rapidly detected. Such improvement would mean overcoming existing barriers to communication; a recent survey found that physicians and veterinarians communicate little about zoonotic issues (*38*). Also, an adequate surveillance network should be developed to detect unusual health events in animal populations. Data on usual trends is missing for most animal species that could be potential sentinels. Whether public health resources can be committed to gathering such baseline data remains an open question.

Second, the results of this review indicate that active surveillance of animal populations, including wildlife and companion animals, could fill a critical need in the aftermath of an attack involving certain bioterrorism agents by helping identify persistent sources of infection in the environment. Third, better approaches for intervention are needed to be able to stem the propagation and amplification of an introduced biological warfare agent into a wild or domestic animal population. The US experience with West Nile virus reflects the difficulties of controlling an emerging zoonotic threat as it spreads through animal populations (*39*).

Finally, the results of this review point out the need for additional research to fill knowledge gaps about animals as sentinels of human disease threats, including data on relative susceptibilities and exposure pathways for animal species living near human populations. Concrete steps could include establishment of surveillance veterinary clinics in strategic areas with incentives for practitioners to report unusual events. Another approach would be to make greater use of electronic databases of animal diseases such as those used by the Banfield Clinics, a nationwide chain of veterinary practices. Similar efforts could be useful with wildlife populations.

Such steps would foster ongoing communication between community practitioners and regional public and private veterinary diagnostic laboratories to establish baseline disease incidence trends and algorithms to identify outbreaks. Common links or web-based interfaces should be developed to integrate human and animal disease surveillance information. Reporting systems for wildlife professionals and the public should be created, and their use should be encouraged to document unusual disease events and die-offs. Another constructive step would be to improve the capacity of existing veterinary rapid-response teams, which exist in many states, to carry out active surveillance with animal populations as well as to improve the coordination of veterinary diagnostic laboratories. Again, barriers to funding and cooperation between animal and human health agencies need to be addressed. In the past, these have hampered efforts to have a coordinated approach to collection of animal surveillance data). In addition, state-based efforts would need to be coordinated on a regional and national scale. The growing awareness that animal health and human health are inextricably linked, however, makes cooperation between human and animal health professionals imperative to strengthen the evidence base that will allow for rational use of animal data in public health decision-making.
